# Perspectives in melanoma: meeting report from the “Melanoma Bridge” (December 5th–7th, 2019, Naples, Italy)

**DOI:** 10.1186/s12967-020-02482-x

**Published:** 2020-09-07

**Authors:** Paolo A. Ascierto, Igor Puzanov, Sanjiv S. Agarwala, Christian Blank, Richard D. Carvajal, Sandra Demaria, Reinhard Dummer, Marc Ernstoff, Soldano Ferrone, Bernard A. Fox, Thomas F. Gajewski, Claus Garbe, Patrick Hwu, Roger S. Lo, Georgina V. Long, Jason J. Luke, Iman Osman, Michael A. Postow, Ryan J. Sullivan, Janis M. Taube, Giorgio Trinchieri, Hassane M. Zarour, Corrado Caracò, Magdalena Thurin

**Affiliations:** 1 Unit of Melanoma, Cancer Immunotherapy and Innovative Therapy, Istituto Nazionale Tumori IRCCS “Fondazione G. Pascale”, Via Mariano Semmola, 80131 Naples, Italy; 2 Roswell Park Comprehensive Cancer Center, Buffalo, NY USA; 3 Temple University School of Medicine, Philadelphia, PA USA; 4 Netherlands Cancer Institute, Amsterdam, The Netherlands; 5 Columbia University Irving Medical Center, Herbert Irving Comprehensive Cancer Center, New York, NY USA; 6 Department of Radiation Oncology, Weill Cornell Medicine, New York, NY USA; 7 Department of Pathology and Laboratory Medicine, Weill Cornell Medicine, New York, NY USA; 8 Department of Dermatology, University of Zurich Hospital, Zurich, Switzerland; 9 Roswell Park Comprehensive Cancer Center, Jacobs School of Medicine and Biomedical Sciences, State University, Buffalo, NY USA; 10 Department of Surgery, Massachusetts General Hospital, Harvard Medical School, Boston, MA USA; 11 Earle A. Chiles Research Institute, Robert W. Franz Cancer Research Center, Providence Cancer Institute, Portland, OR USA; 12 Department of Pathology, University of Chicago, Chicago, IL USA; 13 Department of Medicine (Section of Haematology/Oncology), University of Chicago, Chicago, IL USA; 14 Center for Dermatooncology, Department of Dermatology, Eberhard Karls University, Tübingen, Germany; 15 Department of Melanoma Medical Oncology, Division of Cancer Medicine, Anderson Cancer Center, Houston, TX USA; 16 Jonsson Comprehensive Cancer Center, David Geffen School of Medicine at UCLA, Los Angeles, CA USA; 17 Melanoma Institute Australia, The University of Sydney and Royal North Shore and Mater Hospitals, Sydney, Australia; 18 Medicine University of Chicago, Chicago, IL USA; 19 The Interdisciplinary Melanoma Program, New York University Langone Medical Center, NYU Grossman Medical School, New York, NY USA; 20 Memorial Sloan Kettering Cancer Center and Weill Cornell Medical College, New York, NY USA; 21 Melanoma Program, Mass General Cancer Center, Boston, MA USA; 22 Division of Dermatopathology, Johns Hopkins University School of Medicine, Baltimore, MD USA; 23 Laboratory of Integrative Cancer Immunology, Center for Cancer Research, National Cancer Institute, National Institutes of Health, Bethesda, MD USA; 24 Hillman Cancer Center, University of Pittsburgh, Pittsburgh, PA USA; 25 Department Melanoma, Soft Tissue, Muscle-Skeletal and Head-Neck, Istituto Nazionale Tumori IRCCS “Fondazione G. Pascale”, Naples, Italy; 26 Cancer Diagnosis Program, Division of Cancer Treatment and Diagnosis, NCI, Bethesda, MD USA

**Keywords:** Melanoma, Immunotherapy, Anti-PD-1, Anti-CTLA-4, Target therapy, Biomarkers, CAR-T, BRAF inhibitor, MEK inhibitor, Adjuvant, Neoadjuvant, Combination strategies

## Abstract

The melanoma treatment landscape changed in 2011 with the approval of the first anti-cytotoxic T-lymphocyte-associated protein (CTLA)-4 checkpoint inhibitor and of the first BRAF-targeted monoclonal antibody, both of which significantly improved overall survival (OS). Since then, improved understanding of the tumor microenvironment (TME) and tumor immune-evasion mechanisms has resulted in new approaches to targeting and harnessing the host immune response. The approval of new immune and targeted therapies has further improved outcomes for patients with advanced melanoma and other combination modalities are also being explored such as chemotherapy, radiotherapy, electrochemotherapy and surgery. In addition, different strategies of drugs administration including sequential or combination treatment are being tested. Approaches to overcome resistance and to potentiate the immune response are being developed. Increasing evidence emerges that tissue and blood-based biomarkers can predict the response to a therapy. The latest findings in melanoma research, including insights into the tumor microenvironment and new biomarkers, improved understanding of tumor immune response and resistance, novel approaches for combination strategies and the role of neoadjuvant and adjuvant therapy, were the focus of discussions at the Melanoma Bridge meeting (5–7 December, 2019, Naples, Italy), which are summarized in this report.

## Introduction

The melanoma treatment landscape changed in 2011 with the approval of the first anti-cytotoxic T-lymphocyte-associated protein (CTLA)-4 checkpoint inhibitor and of the first BRAF-targeted monoclonal antibody, both of which significantly improved overall survival (OS). Since then, improved understanding of the tumor microenvironment (TME) and tumor immune-evasion mechanisms has resulted in different approaches to targeting and harnessing the host immune response. The approval of new immune and targeted therapies has further improved outcomes for patients with advanced melanoma using various approaches with distinct modes of action including single or combination of immunotherapy agents, and other combination modalities are also being explored such as chemotherapy, radiotherapy, electrochemotherapy and surgery. In addition, different strategies of drugs administration including sequential or combination treatment may potentially be associated with clinically relevant improvement of outcome.

However, better understanding of resistance mechanisms and reasons why some patients do not respond, or relapse is needed. Treatment strategies in the clinical management of melanoma are evolving in order to address these challenges. Approaches to overcome resistance and to potentiate the immune response are being developed. Increasing evidence emerges that tissue and blood-based biomarkers can predict the response to a therapy. Predictive biomarkers can be considered for patients stratification or selection of patients who will most likely achieve favorable clinical outcome.

The latest findings in melanoma research, including insights into the tumor microenvironment and new biomarkers, improved understanding of tumor immune response and resistance, novel approaches for combination strategies and the role of neoadjuvant and adjuvant therapy, were the focus of discussions at the Melanoma Bridge meeting (5–7 December, 2019, Naples, Italy), which are summarized in this report.

## Melanoma Bridge opening session

### Biomarkers for immunotherapy of cancer

The progress in fully realizing the potential of biomarker-driven assignment for anticancer approaches in immune oncology (IO) requires the development and implementation of novel clinical-grade biomarkers able to guide the selection of a single therapy agent or combination of drugs with complementary mechanisms of action targeting multiple mechanisms of response as well as of immune escape.

Biomarkers for immunotherapy require comprehensive approaches that encompass the complexity of the immune system and tumor biology which cannot be addressed using a single analyte biomarker. Therefore, investigation of the biology and genomics of both the tumor and the host immune system is critical to recognize potential biomarkers. The availability of novel platforms and technologies should facilitate the integration of the molecular features of the tumor and the host factors for the development of multiplex profiles to guide personalized treatment in the future. However, before a candidate biomarker and/or new technology can be used in a clinical setting, rigorous steps to demonstrate the analytical and clinical validity of the biomarkers are required. The challenges to overcome include, the inherent complexity of the assays and independent protocols that result in high data variability and poor reproducibility across sites and studies as well as the requirements for highly specialized bioinformatics expertise for data interpretation and integration of multi-omics data.

In recognition of these challenges, in 2017 the US National Cancer Institute (NCI) at National Institutes of Health (NIH) has established the Cancer Immune Monitoring and Analysis Centers-Cancer Immunologic Data Commons (CIMAC-CIDC) Network. The Network is composed of four laboratories (CIMACs) and a biomarker data storage/access platform (CIDC), to enable systematic analysis and integration of biomarkers associated immunotherapy clinical trials. The overall goals for the CIMAC-CIDC Network are to conduct correlative studies focusing on biomarkers of response and resistance in NCI-supported early-phase immunotherapy trials by offering a wide range of validated analyses using state-of-the-art methods. The network will also facilitate innovative research for new biomarkers, capitalizing on recent advances in immune profiling platforms and technologies, provide centralized bioinformatics resources for data collection and integration across trials, and establish a biomarker database.

Biomarker assays are categorized at tier one, which are broadly recommended for most trials and have harmonized SOPs available across the Network sites including, multiplex immunohistochemistry (mIHC) and immunofluorescence (mIF) panels, mass cytometry (CyTOF), RNA sequencing, whole exome sequencing and T cell receptor (TCR) analysis. Tier two and three assays constitute experimental approaches for which usage depends on the specific trial. In 2018, Partnership for Accelerating Cancer Therapies (PACT) was launched as a public–private partnership with 12 leading biopharmaceutical companies, the NIH, the Food and Drug Administration (FDA), and the Foundation for NIH (FNIH). PACT supports comprehensive immunoprofiling using CIMACs validated assays for IO biomarkers for response to treatment in the industry sponsored immunotherapy trials.

Harmonization of laboratory specific protocols with SOPs and assay performance benchmarks which is the goal of the CIMACs-CIDC Network is necessary to overcome variability of methods and data collection in both academic and industrial laboratories and enable objective interpretation and comparison across different studies and multiple sites. Assay harmonization is an essential component of developing a biomarker database for secondary analyses to accelerate optimization of immunotherapies and the potential success of new immunotherapy agents and combinations.

## Session—melanoma as a model system

### Harnessing the gut microbiome to optimize cancer therapy

The activity of the commensal microbiota significantly impacts human health and has been linked to the development of many diseases, including cancer. Recent study demonstrated that the composition of the gut microbiome can influence the effect of cancer therapy by modulating the antitumor immune response efficacy. Several recent studies have established that the composition of the gut microbiome modulates the efficacy of anti-PD-1 therapy [1–3]. Microbiota taxonomic identification in cancer patients may be affected by geography, disease and sequencing technology. Moving forward, there is a need for a deeper understanding of the underlying biological mechanisms that link specific bacterial strains to host immunity because microbial species associated with anti-PD-1 response in different patients are not always the same across different cohorts. Integrating microbiome effects with other tumor and host factors regulating immunotherapy responsiveness could facilitate optimization of therapeutic outcomes.

Even though there is increasing evidence that the microbiome has a role in influencing the response to therapy, it is not yet known what constitutes a favorable microbiome composition or whether gut microbiota can be altered to improve therapeutic response. However, it opens the exciting possibility to improve efficacy by manipulating the gut flora and various strategies to target the microbiome have been suggested, including antibiotics, probiotics, prebiotics, diet, oral bacterial formulations and fecal microbial transplant (FMT).

FMT approach delivers a fecal microbiota transplant (FMT) and the promise to transfer its beneficial effect. For example, fecal samples could be prepared from anti-PD-1 responders that show a favorable composition of commensal bacteria, then transplanted endoscopically or prepared for oral delivery into patients who are anti-PD-1-resistant and show an unfavorable composition of gut microbes. FMT is being assessed in ongoing trials in patients with melanoma who are refractory to anti-PD-1 treatment. In an ongoing study at the University of Pittsburgh, patients who are non-responders to pembrolizumab at 12 weeks are receiving FMT from a responder patient, with promising preliminary results. An approach using “commensal community” approach is a community of bacterial strains from healthy human donor feces that induces interferon (IFN)-γ-producing CD8 T cells improved the therapeutic efficacy of immune checkpoint inhibitors in syngeneic tumor models [4] and other trials using different consortia are ongoing.

Alternatively, beneficial or immune-potentiating bacteria could be prepared as a probiotics and provided as an immunotherapy adjuvant. Another approach is dietary intervention. Habitual diet is a key determinant of the gut microbiota, and the microbiome composition can be rapidly changed by switching from a high-fat, low-fiber diet to a low-fat, high-fiber diet or vice versa.

All of these approaches lack the precision to modulate very specific bacterial populations and may have variable effects depending on the starting state of the commensal community. This variability offers research potential and requires exploiting the host–microbiome interdependency to consider for personalized therapy. Future goals are the discovery of reliable microbiome-related biomarkers for prediction of response and stratification of patients as well as the identification of favorable microbiota for fecal transfer from responder patients or healthy donors. Identification of communities of commensal bacteria that deliver more potent therapy may be important for integrating microbiome effects with other tumor and host factors regulating immunotherapy responsiveness could facilitate optimization of therapeutic outcomes.

### The unsolved issues in treatment of melanoma in the adjuvant setting

Since 2015, several adjuvant treatments for resected stage III–IV melanoma have been approved. Active adjuvant phase III placebo-controlled trials in melanoma include the CheckMate-76 study of nivolumab and the KEYNOTE-716 study of pembrolizumab, with both studies enrolling patients with resected stage IIb/c disease. These patients have similar survival rates as patients with stage IIIa/b and may benefit from adjuvant therapy.

Implementation of the American Joint Committee on Cancer (AJCC) 8th edition staging manual may disrupt analyses of active adjuvant clinical trials. It has been noted that it is difficult to extrapolate and compare the data from one trial to another, complicating interpretation of current and future trial results in resected stage III melanoma [5]. In analysis of the NYU Interdisciplinary Melanoma Program Database which staged all patients according to AJCC-7 and AJCC-8 guidelines, there was significant improvement in prognostic value for stage III with addition of the IIID sub-stage. However, stage IIC continues to have worse prognosis than IIIA in the revised system.

Approval of adjuvant immunotherapies has led to their inclusion as standard of care for patients with high-risk resected stage III–IV disease. Stage III comprises ~ 23% of new cases each year with ~ 21,500 new cases in the USA each year. Clinical trials have very strict inclusion criteria, but these do not apply to patients treated with standard of care in clinical practice so there may be higher potential for toxicity in this patient cohort. Stage IIB/C patients represent around 10% of new cases, and the challenge is to balance clinical benefit with the risk of toxicity. Only one in four stage III patients benefit from adjuvant therapy [6, 7] and only one in eight stage II patients are expected to derive such benefit.

Although, treatment with combined ipilimumab and nivolumab yielded high response rates (RECIST ORR 73%, pCR 45%) substantial treatment-related adverse events (trAEs) (73% grade 3 trAEs) was observed in this cohort. Whereas treatment with nivolumab monotherapy yielded modest responses (ORR 25%, pCR 25%) and low toxicity (8% grade 3 trAEs). Immune correlates of response were identified, demonstrating higher lymphoid infiltrates in responders to both therapies and a more clonal and diverse T cell infiltrate in responders to nivolumab monotherapy [8].

Treatment-related factors associated with response and toxicity are well characterized, but host factors also need to be considered as adjuvant therapy becomes more widely available. These include the tumor molecular features including T cell infiltrate, mutational load, gut microbiome, germline genetics, proteomic biomarkers and possibly ethnicity. Composition and abundance of the gut microbiome was shown to be associated with immune checkpoint inhibitor response [9]. Two autoimmune germline variants as potential biomarkers of anti-CTLA4 or anti-PD1 immune checkpoint inhibitors (ICI) efficacy in melanoma were identified and suggests that underlying genetic susceptibility to autoimmunity may play an important role during ICI treatments. rs1893217 in PTPN2, involved in cytokine signaling, has been associated with colitis, celiac disease, inflammatory bowel, rheumatoid arthritis and type 1 diabetes. Similarly, rs17388568 was mapped to important immune-related genes (IL-2, IL-21 and ADAD1) and associated with allergy, colitis and type 1 diabetes [10]. Ongoing research focused on predicting response and toxicity emphasizes the need for additional studies to optimize treatment regimens and to validate putative biomarkers that will allow patient selection for adjuvant immunotherapy, an important consideration as the number of patients receiving it as a standard of care increases.

### Emerging targets in the tumor microenvironment to improve radiotherapy and immunotherapy combinations

Increasing the numbers and activation of a specific dendritic cell (DC) subset, conventional type 1 DCs (cDC1s), in tumors can potentially increase the responsiveness of cancer patients to immunotherapy. cDC1s initiate de novo T cell responses after migrating to tumor-draining lymph nodes, as well as recruiting T cells, secreting cytokines e.g. IL-12, and presenting tumor antigens in the tumor microenvironment (TME) (PMID: 30352680). Radiation therapy mediates antineoplastic effects not only by cytotoxic and cytostatic mechanisms, but also by modulating both local and systemic immunological function. However, the mechanisms by which radiation induces antitumor T cells remain unclear. Radiation therapy activates a viral defense response pathway, in which cytosolic DNA stimulates secretion of IFN-β by cancer cells following activation of the DNA sensor cGAS and its downstream effector stimulator of interferon genes (STING). Repeat irradiation at doses that induce optimal cytosolic DNA accumulation amplifies IFN-β production, resulting in recruitment and activation of cDC1s, also known as Batf3-dependent DCs, which are essential for priming of CD8+ T cells that mediate systemic tumor rejection (the abscopal effect). However, epigenetic downregulation of cGAs and/or STING in some tumors may preclude the activation of IFN type I by radiation therapy (REF: PMID: 29367762). We have recently identified an alternative pathway for the recruitment and activation of cDC1 to irradiated tumors. The balance between pro-inflammatory NAD+ and ATP and immunosuppressive adenosine in the TME regulates immune responses, with the accumulation of extracellular adenosine a strategy used by tumors to escape immunosurveillance [11]. CD73 is an ecto-5-nucleotidase that is essential for the generation of extracellular adenosine from 5-adenosine monophosphate (5-AMP). CD73 is the final enzyme for both the canonical and non-canonical adenosine generation pathways In the canonical pathway CD39 (ecto-nucleoside triphosphate diphosphohydrolase-1; ecto-NTPDase1) catalyzes the phospho hydrolysis of ATP and ADP to AMP. In the non-canonical pathway, CD38 catalyzes the conversion of NAD+ to ADPR and CD203a converts ADPR to AMP (PMID: 27209048). Radiation-induced cell death is associated with release of ATP and NAD+ in the TME, but we found that radiation also induces the upregulation of CD38, CD203a and CD73 on cancer cells. Antibody-mediated blockade or genetic knockdown of CD73 restored the recruitment and activation of cDC1 to irradiated tumors that lacked cGAS expression and were unable to produce IFNγ (PMID: 32047024). CD73 blockade led to increased infiltration and activation of CD8+ T cells and decreased regulatory T cells (Treg) in the tumor, associated with complete tumor regression during dual CD73 and CTLA-4 blockade in combination with irradiation.

Thus, CD73 acts as immune checkpoint that precludes radiation-induced anti-tumor T cell activation. High levels of soluble CD73 at baseline have been associated with poor OS and PFS in metastatic melanoma patients treated with nivolumab [12]. Blocking CD73-dependent adenosine-mediated immunosuppression may have the potential to reinstate anti-tumor immunity and synergize with radiotherapy and immune checkpoint blockade to improve tumor control in patients.

### Immunotherapy-induced anti-cancer responses to the spectrum of “Cancer Antigens” and immune contraction

For over 30 years, immune responses against shared cancer antigens have provided evidence of therapeutic activity in preclinical and clinical studies. There is a coordinated B and T cell response to cancer and evidence that IgG responses can correlate with CD8 T cell response. Observations have indicated that circulating cytotoxic T lymphocytes (CTLs) can recognize and eliminate lung cancer cells presenting endogenous non-mutated peptides [13]. Specifically, they demonstrated that NSCLC patients’ sera were found to have IgG antibodies to dozens of proteins from which peptides that were presented on HLA by the NSCLC tumor cells originate. They show that patients could mount a cytotoxic lymphocyte (CTL) response to nonmutated peptides from the same proteins that were the target of IgG responses, and that these CTL could lyse an HLA-matched allogeneic NSCLC.

These and other data suggest patients have broad anticancer immunity against self-non-mutated epitopes and that IgG antibodies identify targets of CD8 T cell response. It is possible that once ‘re-activated’ with antigen and cytokine, T cells can kill cancer cells.

Correlation between increased CD8+ T cell IFN-γ release and serum IgG binding was also demonstrated by a study in which female BALB/c mice were vaccinated with a combination of an autophagosome-enriched vaccine derived from 4T1 mammary carcinoma along with poly-I:C adjuvant where serum was screened for IgG binding to arrays of peptides containing known mutation sites in 4T1 [14]. Simultaneously, CD8+ T cell cultures were primed with peptides derived from these antigens. These primed T cells were then stimulated to measure recognition of the peptides or live 4T1 cells by IFN-γ release. Vaccinated mice showed elevated antigen specific CD8+ T cell recognition of 4T1 tumor cells and CD8 specific peptides. Antibodies had stronger recognition of neoantigens peptides than autoantigens counterpart peptides which differ by a single amino acid substitution, suggesting a bias for the recognition of certain antigens prior to tumor exposure, which may be due to the tolerance to autoantigens or prior exposure to cross-reactive foreign antigens. There was a correlation between increased CD8+ T cell IFN-γ release and serum IgG binding to individual peptide antigens. These reports suggest that antibody immunosurveillance is occurring in early in cancer patients and that IgG antibody responses can be used to identify proteins that are targeted by T-cell responses. Potentially, the benefits of immune checkpoint blockade may be restricted to tumors with pre-existing immune recognition, thus evaluation of immune status may serve as a biomarker for personalization of immuno-therapy. Furthermore, lack of pre-existing immune response should identify patients for de novo boosting immune response including vaccination or combination with other agents that might improve therapeutic efficacy. The DRibbles vaccine is comprised of autophagosome vesicles that contains more than 100 shared cancer antigens. The effect of vaccination with the first allogeneic human DRibbles vaccine, DPV-001, on IgG responses to proteins overexpressed by many/most cancers is being assessed with promising results.

### The role of melanoma derived exosome in modifying the tumor microenvironment

Exosomes are members of the extracellular vesicle community including micro vesicles and oncosomes that originate from multivesicular bodies and that contain proteins, mRNA, microRNA and DNA and are enriched in tetraspanins, i.e. proteins containing four transmembrane domains. Exosomes are cell-derived nano-meter sized (40–100 nm) particles that have been established to play an important role in cell-to-cell communication [15]. Tumor derived exosomes (TEX) have profound impact on the immediate tumor microenvironment (TME) and they can play a role at distant tissue sites to create a pre-metastatic niche conducive to metastasis and are referred as the tumor “macroenvironment” (TMaE). TEX deliver tolerogenic signals to immune cells, inhibiting immune cell proliferation, inducing apoptosis of activated CD8+ T lymphocytes, interfering with monocyte differentiation and promoting the expansion of regulatory T cells thus inducing immune suppression through a paracrine effect. Interestingly, TEX display certain ligands, such as programmed death-ligand 1 (PD-L1), to produce an endocrine signaling effect to generate a favorable pre-metastatic TMaE extending a distance away from the primary tumor.

Exosomes have been widely studied in the recent years as they were discovered to be a mechanism by which tumor cells enhance progression and metastasis. We have demonstrated that one pathway in creating a pre-metastatic niche is through TEX metabolically reprogramming normal fibroblasts and generating an acidic microenvironment detrimental to immune function. HMEX can reprogram the metabolism of normal stromal fibroblasts by skewing the dominant metabolic process from OXPHOS to aerobic glycolysis, encompassing the Warburg effect and inducing extracellular acidification that has been shown to contribute to a pre-metastatic niche and a state of anergy of CD8+ T lymphocytes. Other pathways include direct engagement of TEX with receptors on immune cells as they express ligands that engage receptors on T cells, including the T cell receptor (TCR) and IL-2 receptor (IL-2R) on T cells. TEX PD-L1 can suppress T cell activation and enhance tumor specific immune suppression. TEX inhibit the IL-2 proliferative response in CD8+ T cells and favor regulatory T cell responses. TEX can also suppress monocyte maturation and generate a monocytic myeloid-derived suppressor cell phenotype (Mo-MDSC) that is favorable to tumor escape from immune recognition. TEX may also promote a pre-metastatic niche at a distant site through blood and lymphatic drainage to promote vascular permeability, immunosuppression and metastasis. Exosome distribution through the vascular and lymphatic systems can allow TEX to enhance the TMaE beyond the immediate TME, extending the seed and soil hypothesis by concluding that the soil may be prepared prior to seeding via cancer exosomes and their miRNA and protein payloads. There, TEX act on normal stroma fibroblasts by translocation of growth factor receptors and metabolic reprogramming using microRNA payloads, promoting a switch from oxidative phosphorylation (OXPHOS) to aerobic glycolysis and an increase in extracellular acidification that contribute to the anergy of CD8+ T cells [16].

TEX are important messengers that enhance tumorigenesis and metastasis. They achieve this through a variety of mechanisms including immunosuppression, and molecular and metabolic reprogramming that create a pre-metastatic niche to facilitate the process of tumor progression. Studies have shown that TEX not only influence the immediate TME, but they also travel to distant tissue sites to establish a host-tumor “macroenvironment” through an endocrine phenomenon.

### Low-dose radiation and CAR T cell-mediated in vitro elimination of melanoma cells

A rapidly emerging immunotherapy approach called adoptive cell transfer (ACT) is based on collecting and using patients’ own immune cells to treat their cancer. There are several types of ACT: TILs, TCRs, and CARs, but, thus far, the one that has advanced the furthest in clinical development is called chimeric antigen receptor (CAR) T-cell therapy. The selection of appropriate target antigens in solid tumors remains challenging for the therapeutic development of safe and effective CAR-T-based therapies. The high expression of B7-H3 and chondroitin sulfate proteoglycan 4 (CSPG4) across multiple tumor types such as prostate, breast, placenta, liver, colon, and lymphoid organs and restricted expression in normal tissues makes them attractive targets for immunotherapy. CSPG4 is a cell surface type I transmembrane protein critical for tumor progression and metastasis. B7-H3 (CD276) is an immune checkpoint from the B7 family of ligands, many of whom interact with known checkpoint markers including CTLA-4, PD-1, and CD28 and it is overexpressed in many cancer types.

Besides identification of a suitable tumor associated antigen (TAA), trafficking of administered CAR T cells to the tumor is another challenge to effective therapy. In addition to the limited trafficking of CAR T cells to solid tumors the mechanisms of resistance against CAR T cell mediated killing includes immunosuppressive TME. Consequently, experimental models to improve innate CAR T cell trafficking via coexpression of chemokine receptors have been developed. Therapeutic activity of CAR-Ts can also be limited due to the rapid tumor escape when the targeted antigen shows heterogeneous expression within the tumor, as recently reported in patients with glioblastoma treated with CAR-Ts specific for EGFRVIII. Increasing evidence suggests that intrinsic characteristics of CAR molecules, such as the epitope recognized by the CAR and the affinity of the mAb from which the CAR is derived, may play a significant role in discriminating antigen recognition in normal versus malignant cells.

BRAF mutation has been shown to downregulate HLA class I and tumor antigen expression on melanoma cells. Radiation upregulated B7-H3 and CSPG4 expression on BRAF wild type human MV3 melanoma cells treated with a BRAF inhibitor. In BRAF-mutated human M21 melanoma cells, radiation induced B7-H3 level but there was limited modulation of CSPG4. Radiation also upregulates HLA-A/B/C on human M21 melanoma cells. Radiation seems to have a higher effect on BRAF inhibitor-resistant cells compared to their BRAF inhibition-sensitive counterparts in terms of tumor antigen upregulation and modulation of pro- and anti-apoptotic molecules. Radiation upregulated B7-H3 expression on human M21-R melanoma cells, resistant to BRAF inhibitors. In addition there was differential in vitro elimination of both BRAF inhibitor sensitive and resistant melanoma cells by B7-H3- or CSPG4-specific CAR T cells.

Radiation can render melanoma cells more sensitive to CAR T cell-mediated lysis through upregulation of the targeted tumor antigen, and imbalance between pro- and anti-apoptotic molecules. At high effector to target cell (E:T) ratio, there is no difference in terms of the cytotoxic effect of CAR T cells against BRAF inhibitor sensitive and resistant melanoma cells. However, at lower E:T ratio, CAR T cells demonstrate more potent in vitro antitumor activity with irradiated BRAF inhibitor resistant melanoma cells.

These data not only support the potential of achieving clinical benefits using CAR T cells in solid tumors but also highlight that their antitumor activity can be enhanced by radiation of target tumor cells.

### The model system for understanding checkpoint inhibitor resistance in cancer

Immune checkpoint inhibitors have revolutionized the treatment of patients with advanced-stage metastatic melanoma, as well as patients with many other solid cancers, yielding long-lasting responses and improved survival. However, a subset of patients who initially respond to immunotherapy, later relapse and develop therapy resistance (termed “acquired resistance”), whereas others do not respond at all (termed “primary resistance”). Primary and acquired resistance are key clinical barriers to further improving outcomes of patients with metastatic melanoma, and the known mechanisms underlying each involves various components of the cancer immune cycle, and interactions between multiple signaling molecules and pathways.

Although there is a large body of literature on response, the specific mechanisms of resistance and biomarkers of resistance have not been well studied. Overcoming therapy resistance requires a thorough understanding of the mechanisms underlying immune evasion by tumors. For an immunotherapy to elicit an efficient antitumor immune response, the cancer immune cycle must be initiated, and the subsequent steps successfully completed. This involves efficient (i) antigen presentation and T-cell activation, (ii) T-cell trafficking and tumor infiltration, and (iii) T-cell killing activity within the tumor microenvironment.

Studies examining possible predictive biomarkers of response to immunotherapy have reported a higher density of preexisting cytotoxic T lymphocytes in tumor biopsies of patients who displayed a greater response to anti-PD-1/PD-L1 immunotherapy, and more significantly, an increased influx of T cells and PD-L1 macrophages early during treatment.

Patients with advanced melanoma with a favorable prognosis tend to be those with low volume disease, normal baseline LDH and a low number of metastatic sites. Tumors with high tumor mutation burden (TMB) and high IFN-γ-gene expression profile (GEP) both exhibit independent predictive value for response to anti-PD-1 therapy, and represents the best tissue-related predictive tool to date, although not clinically validated in prospective trials [17]. The analysis of tumors across 22 tumor types including melanoma from 4 KEYNOTE clinical trials showed that TMB and inflammatory biomarkers (T cell-inflamed GEP and PD-L1 expression) can jointly stratify human cancers into groups with different clinical responses to pembrolizumab monotherapy. With this stratification in mind, we can identify patterns of underlying, targetable biology related to these groups. TMB and inflammatory biomarkers independently predict response and may capture distinct features of neo antigens presence and T cell activation, respectively.

Comprehensive clinical and genomic analysis demonstrated that TMB and IFN-γ expression independently predicted response in 77 melanoma patients treated with anti-PD-1 monotherapy or combined with ipilimumab [18]. However, several patients did not fit this pattern as they either responded despite having low TMB and IFN-γ or had high TMB and IFN-γ but did not respond. These patients are of particular interest in terms of understanding possible mechanisms of resistance.

In the clinic, patients who progress can be categorized into four distinct primary progression groups: homogeneous and generalized with no benefit from therapy (more common with immunotherapy than targeted therapy), primary progression that is heterogenous and solitary/oligometastatic, secondary progression after an initial response (i.e. acquired resistance) that is heterogenous and solitary/oligometastatic, and secondary progression that is homogeneous and generalized (more common with targeted therapy). These clinical definitions can be used in prognostic models.

The resistance occurs when there is failure to induce an effective antitumor immune response, as demonstrated by primary or acquired resistance to immunotherapy in melanoma. These two types of resistance are characterized by several molecular features.

1. Primary resistance patients have elevated levels of baseline serum LDH, low tumor burden and lack of PD-L1 expression in baseline melanoma tissue samples, lack of T-cell infiltration, the absence of PD-1 T cells and PD-L1 macrophages in melanoma biopsies taken early during treatments, insufficient neoantigens and low mutational burden, the presence of an innate anti-PD-1 (IPRES) transcriptional resistance signature, or absence of an interferon signature.
2. Acquired resistance occurs in patients who relapse after exhibiting an initial response to immunotherapy. An example of one such trait is beta-2-microglobulin (B2M), a component of MHC class I molecules that is necessary for their functional expression. The loss of B2M expression was reported in melanoma cell lines from five patients who had been treated with immunotherapy and cytokine-gene therapy. This resulted in a loss of MHC class I expression and, therefore, a subsequent decrease in recognition by CD8þ T cells. JAK1/2 mutations have also recently been identified as genetic markers of acquired resistance to immunotherapy in melanoma that is responsible for cell proliferation, differentiation, cell migration, and apoptosis. Other immune checkpoint markers such as lymphocyte activation gene 3 (LAG-3) and T-cell immunoglobulin and mucin domain 3 (TIM-3, HAVCR2) have also been revealed to interfere with the activity of T cells, resulting in acquired resistance to immunotherapy.

The research approach for molecular characterization of underlying mechanism of resistance requires analysis of biopsies at baseline, early during treatment and at disease progression. However, challenges in using advanced melanoma as a model include heterogeneity of tumors, with samples enriched for subcutaneous and lymph nodes and limited sampling of other sites, quality issues in sampling of metastases. An alternative model that offers the advantages of abundant is homogenous resistant tumor tissue from neo-adjuvant trials. Several trials are ongoing in the neoadjuvant setting with to provide high quality matched clinical data and clear endpoints, with the primary endpoint of pathological complete response (CR) closely correlated with the secondary endpoint of long-term recurrence-free survival (RFS) [19].

### Electrochemotherapy in integrated approach of metastatic cutaneous melanoma

Electrochemotherapy (ECT) is an electroporation (EP)-based technology used for clinical application and research that includes drug delivery. Specifically, ECT is a local and nonthermal tumor ablation modality, which combines the administration of a poorly permeant cytotoxic agent with the local application of electric pulses that induce reversible EP, thus improving drug diffusion into the cells. ECT is performed using either intratumoral or intravenous cytotoxic drug injection, followed by the application of electric pulses locally delivered to the target tumor. ECT can be used when surgery is not an option. ECT has comparable or superior effectiveness over several ablative skin-directed therapies such as over photodynamic therapy, radiotherapy, intralesional therapy, and topical therapy. Also, it can be used for chemotherapy-resistant and radiotherapy-resistant lesions. ECT is suitable for patients with severe comorbidity and/or patients of an advanced age who have already exhausted all other treatments. Indications for its use in melanoma include early cutaneous relapse after surgical treatment and effective treatment of tumor lesions located in the skin or subcutaneous tissue, both primary and metastatic. It has also shown its effectiveness in the case of treating deep-seated tumors. ECT can be also used as an alternative approach or as a palliative treatment after standard therapies (such as surgery, radiotherapy, and chemotherapy) to improve the quality of life for patients, and as neoadjuvant therapy for extensive lesions or to reduce the surgical approach. ECT can provide long-term benefit in terms of curative and palliative treatment for unresectable cutaneous lesions [20]. In a prospective study of 376 patients with superficial metastases, tumor response rate at 60 days was 88% (50% CRs) [21]. ECT was also shown to be a highly effective local treatment for melanoma metastases in the skin in an International Network for Sharing Practices on Electrochemotherapy (InspECT) trial that reported an objective response rate (ORR) of 74%, 1-year OS of 67% and MSS of 74% [22]. Treatment was well tolerated with no serious adverse events. Coverage of deep margins, previous irradiation of the treated area and tumor size (< 3 cm) were positively associated with a CR.

As with radiotherapy, ECT may induce an abscopal effect which suggests the potential for combining with immunotherapy. This may result from releasing of tumor antigens due to ECT treatment and induction of inflammatory response, cytokine production, complement activation, increased MHC class I expression and T cell activation resulting from checkpoint inhibition. Retrospective analysis of 15 patients treated with ipilimumab who received ECT reported a local ORR of 67% of patients (27% CR and a systemic response in 60% of patients [23]. Evaluation of circulating regulatory T cells (Tregs) demonstrated significant differences between responders and non-responders. Overall, treatment was well-tolerated. In 127 melanoma patients treated with ipilimumab, the combination with local peripheral treatment (local irradiation, skin directed ECT or selective internal radiotherapy of liver metastases) significantly prolonged OS (93 vs. 42 weeks with ipilimumab alone) [24]. Immune-related toxicities were not increased by the combination and local peripheral treatment-induced local toxicities were mostly mild. Overall, ECT appears to be feasible and tolerable, with potent anti-tumor activity and a high response rate, and the potential for use with immunotherapy.

ECT is used currently for treatment of cutaneous and subcutaneous lesions, without consideration of their histology. It is also becoming a practical method for treatment of internal, deep-seated tumors and tissues.

### New perspectives in uveal melanoma

Uveal melanoma (UM), a rare subset of melanoma, is the most common primary intraocular malignancy in adults. Despite effective primary therapy, nearly 50% of patients will develop metastatic disease. Outcomes for those with metastatic disease remain dismal due to a lack of effective therapies. Current immunotherapies are less effective in UM than cutaneous melanoma. A meta-analysis in patients with metastatic uveal melanoma treated with anti-PD-1/PD-L1s reported an ORR of only 3.6% and a median PFS of 2.6 months [25]. These poor results were confirmed in another meta-analysis, which showed a median PFS of 2.8 months in patients receiving immunotherapy [26]. Results of single-agent checkpoint blockade for UM have generally been disappointing. Dual checkpoint blockade with ipilimumab and nivolumab in combination has achieved numerically superior outcomes to checkpoint blockade monotherapy; however, outcomes were still comparatively poor. In a phase II, single-arm trial in 50 patients with untreated metastatic UM, ORR was 12%, median PFS 3.3 months and median OS 12.7 months [27]. Treatment-related adverse events were reported in 46 patients and nine patients discontinued treatment.

The differential response to checkpoint blockade between uveal and cutaneous melanoma may, in part, it can be explained by the unique biology and immunology of uveal melanoma that necessitates the development of dedicated management and treatment approaches. The vast majority (85–95%) of uveal melanoma is characterized by activating mutations in genes encoding the G-protein-alpha subunits GNAQ or GNA11, which lead to stimulation of the MAPK and phosphatidylinositol 3-kinase (PI3K)/Akt pathways. Several other genetic alterations have been implicated in the development of uveal melanoma. Inactivating mutations in BAP1, a tumor suppressor gene located on chromosome 3p, are found in approximately 47% of primary uveal melanoma and 84% of metastatic uveal melanoma cases, consistent with the association between BAP1 mutations and poor prognosis. Mutations in splicing factor 3B subunit 1 (SF3B1), involved in pre-messenger RNA splicing, while associated with more favorable prognostic features than BAP1 mutations, are also found in cases of delayed metastasis, with a median of 8.2 years. EIF1AX encodes for eukaryotic translation initiation factor 1A. These mutations are mutually exclusive from BAP1 and SF3B1 and are associated with a longer disease-free survival and a more favorable prognosis

Furthermore, metastatic UM are characterized by the low mutational burden observed in uveal melanoma may partly account for the limited success of immune checkpoint blockade. Moreover, upregulation of immunosuppressive factors such as Indoleamine 2,3-dioxygenase (IDO1) and T cell immunoreceptor with Ig and ITIM domains (TIGIT) may contribute to treatment resistance and suggests a role for combination immune therapies targeting these additional factors. There is a significant decrease in PD-1-positive lymphocytes and lower levels of PD-L1 in metastatic uveal melanoma compared with cutaneous melanoma metastases. Tumors from metastatic UM patients also show a lower rate of tumor-infiltrating lymphocyte (TIL) expansion compared with metastatic cutaneous melanoma [28].

However, while uveal and cutaneous melanoma diverge in many features, phenotypic commonalities such as expression of the melanoma-associated antigen gp100 remain. Data from The Cancer Genome Atlas (TCGA) show that transcript expression of gp100 is observed in both with more uniformly high expression of gp100 transcript in primary uveal melanoma compared with compared primary cutaneous melanoma. Tebentafusp (IMCgp100) is a bispecific biologic in development by Immunocore, comprising targeting and effector moieties. The targeting end constitutes a soluble T cell receptor (TCR) that recognized the melanocyte-associated antigen glycoprotein 100 (gp100) presented in the context of HLA-A2, which is expressed in approximately 50% of patients with uveal melanoma, and the effector end includes an anti-CD3 single chain variable fragment (scFv). In vitro, IMCgp100 redirects a potent T cell-mediated immune response toward gp100 positive melanoma cells. In a phase I trial, 3/17 (18%) patients with metastatic uveal melanoma achieved a PR and 11/17 (65%) achieved disease control for ≥ 16 weeks. OS rates at 1 year of 74% (95% CI 48–88) were also achieved [29]. Two potential explanations for the apparent beneficial effect of tebentafusp in uveal melanoma are that gp100 expression is particularly high in this tumor type and that recruiting T cells to antigen-positive sites and inducing an inflammatory response in the presence of a relatively non-T cell-inflamed genetic signature might help overcome this barrier. The therapeutic advances that have translated to improved patient survival in cutaneous melanoma have unfortunately not yielded similar benefits in advanced uveal melanoma. However, ongoing efforts seek to optimize the efficacy of targeted therapy and immunotherapy in both the adjuvant and metastatic setting.

## Session—mechanism of resistance and drivers of response

### Translational research in the metastatic melanoma: recent results

Cell invasion through the basement membranes is crucial during morphogenesis and cancer metastasis. The basement membrane is a dense, highly cross-linked, sheet-like extracellular matrix that underlies all epithelia and endothelia. During development of metastatic disease, cells cross the basement membrane to disperse and enter new tissues. This complex invasive process depends on a coordinated network of polarization, the production of matrix metalloproteinases, breaching of the basal membrane and the formation of invadopodia-like structures. The transmembrane migration of cancer cells recapitulates the penetration of epithelial cells during development.

Models to study basal membrane breaching include chick chorioallantoic membrane, drosophila imaginal discs, studies of leukocyte transmigration in vertebrates and intravital imaging studies in murine tumor models and anchor cell invasion in the nematode *Caenorhabditis elegans*. Anchor-cell invasion in *C. elegans* is a simple and attractive model of regulated cell-invasive behavior.

Importantly, the pre-replication complex including cyclin-dependent kinase CDKN2A is an essential component during basal membrane disruption. Several components of the DNA pre-replication complex are required for anchor cell invasion. Three genes required for normal anchor cell invasion encode cell cycle regulators: cell division cycle 6 (CDC6) ATP binding protein is an essential component of the DNA pre-replication complex, CYD-1 encodes the only cyclin D homolog in *C. elegans* and cyclin-dependent kinase 12 (CDK12) encodes the CDK required for activation of RNA polymerase II. The pre-replication complex controls actomyosin polarity and pro-invasive gene expression in the G1-arrested anchor cell.

The acquisition of invasive behavior also marks a critical transition during melanoma progression [30]. The study provides direct evidence that this ability is promoted by increased cellular motility and migration of neoplastic melanocytes from the epidermis into the subjacent dermis that results from CDKN2A loss. The most common acquired genetic change distinguishing precursor lesions, such as melanocytic nevi or melanoma in situ (MIS), from invasive melanomas is loss of the CDKN2A locus. The CDKN2A locus encodes two gene products—p14ARF and p16INK4A—each under transcriptional regulation by independent promoters and each with distinct tumor suppressive functions. The loss of p16INK4A promotes melanocyte motility and the invasive and metastatic capacity of melanoma cells through the transcriptional activation of BRN2, a transcription factor previously associated with melanocytic invasive programs during both development and disease was demonstrated [31]. Loss of p16INK4A promotes melanocyte motility and the invasive and metastatic capacity of melanoma cells through the transcriptional activation of BRN2, a transcription factor previously associated with melanocytic invasive programs during both development and disease. Targeting of the pre-replication complex is a promising approach for chemoprevention of melanomas and other cancers.

### Intrinsic tumor genomic and metabolic factors leading to immunoresistance

**Fig. 1:**
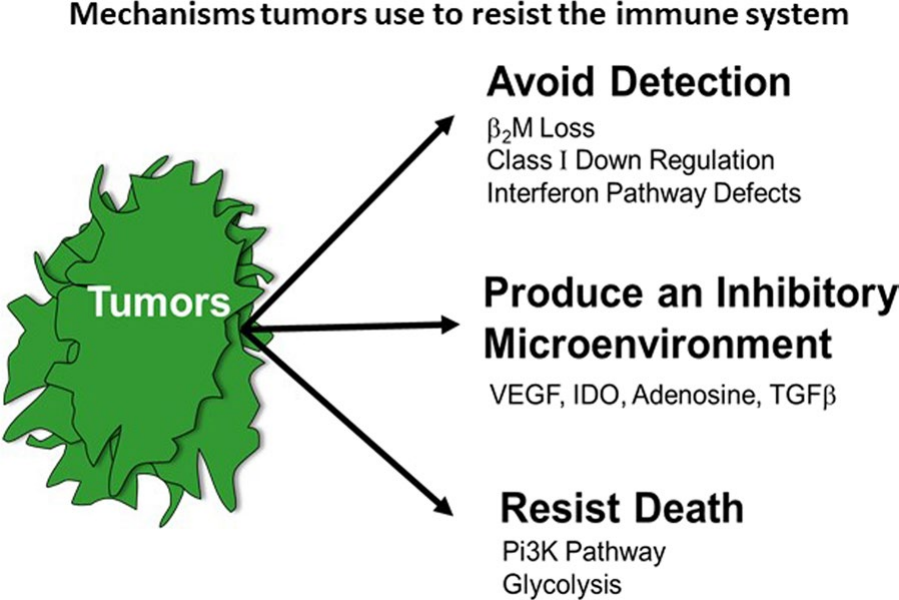
Mechanisms tumors use to resist the immune system: avoided detection, production of inhibitory microenvironment, death resistance

Significant progress has been made in the field of cancer immunotherapy however, durable responses are only achieved in a subset of patients, and currently there is very limited ability to predict whether a patient is likely to respond to immunotherapy. Recently, several studies have elucidated some of the tumor intrinsic molecular mechanisms of resistance to immunotherapy. Tumors use various mechanisms to evade the immune system that involve avoiding detection, promoting an immunosuppressive microenvironment, and resisting cell death. For example, tumor cells can avoid detection through B2M loss and class I down regulation (Fig. 1).

RNA-binding protein MEX3B was identified as a candidate protein whose overexpression in melanoma cells decreased their susceptibility to killing by autologous TIL in vitro suggesting that it mediates resistance to cancer immunotherapy. Overexpression of MEX3B in melanoma cells decreased IFN-γ release by autologous TILs and downregulated HLA-A expression [32]. Downregulation of HLA-A expression by MEX3B is a novel mechanism for tumor cells to evade attack by T cells. Analysis of anti-PD-1 treated melanoma patient tumor samples suggested that higher MEX3B expression is associated with resistance. Our findings have the potential to lead to the development of therapeutic strategies targeting MEX3B in hope of overcoming immunoresistance and achieving better clinical outcomes for patients with melanoma treated with immunotherapy.

Tumor cells can also produce a microenvironment that inhibits immune cells. Tumor induced immunosuppression by transforming growth factor‐β (TGF‐β), a cytokine with pleiotropic effects on cell growth and differentiation is known mechanism of immunotherapy resistance. We hypothesize that truncating the intracellular domain of the TGF‐β receptor will abrogate immunosuppressive signalling through this pathway, which may augment immunotherapy effectiveness. Preclinical studies showed safety and efficacy of virally transducing T cells with TGF‐DNRII and improved proliferation and function of T cells. Treatment with TILs engineered to express TGF-β DNR and nerve growth factor receptor (NGFR) (which is truncated to render it incapable of signalling, serves as a control) resulted in best imaging response by irRC that was PD/SD/PR in 1/5/1 pts with DCR of 86%. All 5 SD patients have had disease reduction of 12–48% with responses ongoing. The PR patients continues to respond 15 months after therapy. There was no added toxicity from the gene modified TIL with toxicities attributed to lymphodepletion and IL‐2. Genetic modification of TIL with TGF‐DNRII is feasible to generate, safe to administer and has demonstrated efficacy in malignant melanoma. [8].

Loss of the tumor suppressor gene phosphatase and tensin homolog (PTEN) inhibits T cell infiltration into tumors and has been found to correlate with resistance to anti-PD-1 in melanoma patients [33]. We recently demonstrated that loss of PTEN impedes trafficking of effector T cells to tumors, reduces the sensitivity of melanoma cells to T cell mediated killing, and correlates with inferior outcomes in patients treated with immune checkpoint blockade. Treatment with a selective PI3Kβ inhibitor improved the anti-tumor activity of anti-PD-1 in a genetically engineered PTEN loss tumor model.

Recent studies also identified glycolysis as a candidate pathway of resistance. Immuno-resistant PTEN-silenced tumors display increased glycolytic activity [34]. Upregulated expression levels of glycolysis-related genes were associated with poor T-cell infiltration in melanoma and NSCLC tumor samples. Overexpression of glycolysis-related enzymes impaired T cell killing of tumor cells, whereas inhibition of glycolysis enhanced T cell-mediated antitumor immunity in vitro and in vivo. Inhibition of glycolysis enhanced efficacy of adoptive T-cell therapy (ACT) and increased glycolytic activity was detected in cell lines from melanoma patients non-responding to ACT. Reduced expression of IRF1 and CXCL10 immunostimulatory molecules was observed in highly glycolytic melanoma cells. These findings indicate that increased tumor-intrinsic glycolytic activity is associated with poor tumor migration of T cells and reduces susceptibility of tumors to T cell induced apoptosis. Critical role of tumor intrinsic glycolysis in modulating T cell-mediated antitumor activity may lay foundation for the development of glycolysis inhibitors to improve the effectiveness of ACT for cancer treatment.

### Tumor mutation burden and liquid biopsies: helpful for treatment decisions?

Localized and advanced cancers may generate circulating tumor cells and circulating cell free tumor DNA (ctDNA) that can be detected and quantified from peripheral blood samples (liquid biopsy). For melanoma patients, liquid biopsy results may serve as novel predictive biomarker to guide therapeutic decisions, particularly in the context of mutation-based targeted therapies. Tumor mutation burden (TMB) is a potential biomarker for immunotherapy response and early measurement of circulating tumor ctDNA can help to detect treatment failure to immunotherapy. However, it has not yet been clarified how TMB and ctDNA can be used to estimate response to combined CTLA-4 and PD-1 antibody therapy in metastatic melanoma. Novel detection technologies have significantly improved the sensitivity and specificity of the ctDNA detection assays as due to the low abundance of ctDNA, its detection requires highly sensitive and specific techniques. The analysis of ctDNA provides three parameters that may be correlated with clinical outcome: (1) the total quantity of ctDNA in the sample, (2) the molecular fingerprint of the ctDNA by mutation detection, and (3) the quantification of mutant copy numbers. Three of the most frequently used techniques are digital PCR (dPCR), BEAMing and allele-specific ligation PCR (LPCR). The clinical implications of liquid biopsy in routine diagnostic testing of melanoma patients is promising. In a prospective biomarker study, 35 melanoma patients treated with ipilimumab and nivolumab were assessed using a tumor panel of 710 tumor-associated genes followed by repeat liquid biopsies [35]. Tumor mutational burden (TMB) was higher in responders than in non-responders and TMB > 23.1 Mut/Mb (TMB-high) was associated with a melanoma-specific survival (MSS) benefit compared to TMB ≤ 23.1 Mut/Mb (TMB-low or TMB-intermediate). Liquid biopsies every 3–4 weeks were taken for analysis of ctDNA using targeted gene panel for sequencing. At first follow-up 3 weeks after treatment initiation, increased ctDNA concentration was observed more often in non-responders and a > 50% decrease in ctDNA was significantly associated with response to combined immunotherapy. A > 50% increase in ctDNA, or detectable/increasing ctDNA at first follow-up were significantly associated with worse OS while patients with high TMB showed a trend towards prolonged survival. OS was worse in patients with TMB ≤ 23.1 Mut/Mb and either ctDNA increase/detectable or ctDNA increase of > 50% at first follow-up. Liver metastases also had a significant negative impact on response and there was a trend towards lower response rate with elevated lactate dehydrogenase (LDH), previous targeted therapy and PD-L1 expression < 1%.

Other reports have suggested ctDNA can be predictive of response to anti-PD-1 therapy in metastatic melanoma. Longitudinal assessment of ctDNA in 76 patients receiving treatment with PD-1 inhibitors was an accurate predictor of response, PFS and OS [36].

The use of liquid biopsy in routine diagnostic testing remains limited due to the following challenges: (1) clonal heterogeneity, e.g. the detected ctDNA might not represent the predominant tumor clone, thus the progression of the major clone might be overlooked, (2) not defined origin of ctDNA (primary, metastatic, or premalignant lesion; or (3) different cancers developing during the course of disease/treatment.

Localized and advanced cancers may also generate circulating tumor cells (CTCs) that are detectable in the blood. CTCs show promising results in establishing the tumor response to chemotherapy and in the prognosis of cancer patients. Numerous assays for the direct detection of CTCs have been developed that allow for the analysis of CTCs at the single-cell stage in the peripheral blood. In a study of 84 patients with malignant melanoma, a combined analysis of ctDNA and CTCs predicted relapse earlier than imaging and was more accurate than serum LDH or S100 in a subset of patients [37]. Overall, 32% of patients were CTC-positive. An increase in CTC-positive patients was detected with increased tumor staging. Analysis of CTCs count and ctDNA < 150 bp appear to be promising to predict tumor burden in melanoma patients, as negative values may indicate a complete remission.

Although the clinical relevance of CTCs and ctDNA for disease monitoring in patients with metastatic disease is well known and currently entering routine clinical diagnostic use, the role of these biomarkers in early-stage cancer patients remains to be investigated.

### Primary and secondary mechanisms of immunotherapy resistance

The immune system has the potential to recognize and eliminate tumor cells, and failed immune surveillance contributes to cancer development. Many immunotherapeutics, including checkpoint blockade therapy, harness the endogenous antitumor immune response. While clinical benefit can be profound, some patients show primary resistance, whereas others experience clinical response and then develop secondary resistance. General mechanisms include adaptive evasion, allowing the tumor to establish equilibrium with an existing T cell infiltrate, and innate evasion, in which T cells and other immune cells are excluded from the tumor microenvironment.

Clinical studies have indicated that tumors lacking a baseline infiltrate of activated T cells typically fail to respond to checkpoint blockade therapy. For T cell-inflamed tumors, multiple mechanisms have been implicated in defective T cell-mediated tumor elimination. T cells that do effectively home to tumor metastases (based on chemokine gradients and activated vascular endothelial cells) may become dysfunctional, pointing toward immunosuppressive mechanisms in the tumor microenvironment. T cell anergy due to insufficient B7 co-stimulation, extrinsic suppression by regulatory cell populations, inhibition by ligands such as programmed death ligand-1 (PD-1), metabolic dysregulation by enzymes such as indoleamine-2,3-dioxygenase (IDO), and the action of soluble inhibitory factors such as transforming growth factor-β (TGF-β) have all been reported to contribute to this suppressive microenvironment. This suggests the paradigm of restoring CD8+ T cells already in the tumor through blocking negative regulation.

Gene expression signatures based on the T cell-inflamed phenotype in the tumor microenvironment are associated with the presence of an adaptive immune response and clinical benefit from PD-1/PD-L1 blockade [38]. This signature was developed on the NanoString nCounter gene expression system (NanoString Technologies, Inc., Seattle, WA) in the context of pembrolizumab treatment as a pan-tumor determinant of response to PD-1 directed therapy. Samples were obtained at baseline from patients undergoing treatment with pembrolizumab in clinical trials of multiple distinct tumor types in a rigorous stepwise validation of the hypothesis that immune-related gene signatures can enrich for clinical response to PD-1 checkpoint blockade.

Another potential biomarker relevant to a broad spectrum of tumors is measurement of total tumor mutation burden (TMB). The correlation of TMB and response to checkpoint inhibitors was demonstrated in lung cancer, which has a broad range of nonsynonymous mutations within the tumor. Overall, tumor types with higher median mutation burden tend to be more responsive to checkpoint inhibitors than tumors that harbor few mutations. The earliest successes of checkpoint inhibitors were in melanomas and non-small cell lung cancers, two tumor types that can have high mutation burden due to mutagen exposure (UV light and tobacco smoke).

Multiple tumor cell-intrinsic oncogenic events may contribute to primary or acquired resistance to immunotherapy. The WNT-β-catenin pathway appears to be associated with a non-T cell-inflamed TME, as β-catenin-positive tumors had minimal T cell infiltration and have been shown to be more resistant to checkpoint blockade therapy. Specifically, tumor cell-intrinsic β-catenin activation prevents host anti-tumor immune response by failure to recruit Batf3-leneage DCs, which are involved in both the priming phase and the effector phase of anti-tumor immune response [39]. Batf3-DCs in the tumor microenvironment (TME) are also necessary at the time of anti-PD-L1 therapeutic effect. Batf3+ DCs clustering with CD8+ T cells in tumors correlate with a positive IFN-γ gene-signature.

Beyond β-catenin, gene deletions and loss-of-function mutations of the tumor suppressor phosphatase and tensin homolog (PTEN) have also been associated with poor T cell infiltration in the tumor microenvironment in metastatic melanoma. Loss of PTEN, which leads to increased activation of the phosphatidylinositol 3-kinase (PI3K)–Akt pathway, has been associated with primary and secondary resistance to PD-1 blockade in melanoma [40]. Features of T cell-inflamed TME can serve as predictive biomarkers for response to anti-PD-1-based immunotherapies. Primary resistance to checkpoint blockade in most cases is associated with the absence of a T cell inflamed TME. Non-T cell-inflamed tumors have no shortage of antigens, but lack Batf3-lineage DCs. T cell exclusion from the TME can be mediated by β-catenin, PTEN loss, and other oncogenic events. Secondary resistance to immunotherapies can arise upon selection for new oncogenic variants that mediate T cell exclusion. Two oncogenic events linked to poor T cell infiltration and secondary immunotherapy resistance are tumor cell-intrinsic β-catenin pathway activation and PTEN loss-of-function mutation or deletion.

### Next target for immune checkpoint blockade: Tim-3 in cancer immunotherapy

Multiple negative immunoregulatory pathways impede T cell-mediated tumor destruction in the tumor microenvironment (TME), contributing to the paradoxical coexistence of TA-specific CD8+ T cells and tumor progression in cancer patients. Among them, inhibitory receptors (IR) like PD-1 and CTLA-4 play a critical role in dampening T cell functions. Immunotherapies with immune checkpoint inhibitors directed against these immunoregulatory pathways provide long-term clinical benefits to patients with a growing range of solid tumors. Exhausted T cells upregulate a large number of these receptors, including PD-1, CTLA-4, T cell immunoglobulin domain and mucin domain containing-3 (Tim-3) also known as Hepatitis A virus cellular receptor 2 (HAVCR2), lymphocyte activation gene 3 (LAG-3), and T cell tyrosine-based inhibitory motif domain (TIGIT). These inhibitory receptors are expressed by a significant number of tumor antigen-specific T cells, with ligands highly expressed in the TME. There is evidence of additive/synergistic effects on tumor antigen specific CD8+ T cell expansion, supporting the concept of dual blockade through combined therapeutic approaches to improve the efficacy of the treatment.

Tim-3 is an important negative regulator of innate and adaptive immunity. In cancer, PD-1^+^Tim-3^+^ CD8+ T cells are dysfunctional/exhausted. Notably, TIM-3 appears to be an adaptive mechanism of resistance to PD-1 blockade in mouse lung tumor models. Besides CD8 TILs, TIM-3 is also expressed on Tregs and multiple innate immune cell types, including NK cells and APCs. Tim-3+ Fox3+ Tregs, represent a subset of highly activated/suppressive Tregs, which also express high-level PD-1, CTLA-4, and LAG-3. The binding of Tim-3 to phosphatidylserin promotes the uptake of apoptotic cells and cross-presentation of antigen by dendritic cells, which constitutively express high-level Tim-3. Multiple ligands can bind to Tim-3, including galectin 9, Phosphatidylserin, CEACAM-1 and HMGB1. The role of these ligands in mediating Tim-3-mediated immunosuppression in human cancers remain to be elucidated.

Preclinical data have shown that dual blockade of PD-1 and TIM-3 augments tumor antigen-specific cell responses in vitro with evidence of reduced tumor growth in vivo. These data suggest that the PD-1 and TIM-3 pathways promote T cell dysfunction in a non-redundant fashion. They also support that dual blockade of PD-1 and TIM-3 reinvigorates effector T cell responses more potently than single blockade. In a murine model of breast cancer, TIM-3 expression appeared to regulate the function of CD103+ DCs through CXCL9 expression [41]. Anti-TIM-3 antibody indirectly enhanced a CD8+ T cell response during chemotherapy. Anti-TIM-3 or anti-galectin 9 antibody increases CXCL9 expression by DCs. Therapeutic efficacy was ablated by CXCR3 blockade, Batf3 deficiency, or Irf8 deficiency.

Dual blockade of PD-1 and Tim-3 has shown evidence of clinical activity with manageable toxicity in PD-1 refractory NSCLC and melanoma patients [42]. Objective responses observed were in PD-L1-positive (TPS ≥ 1%) patients, indicating the potential for biomarker enrichment. This combination is being further assessed in several clinical trials.

### Histopathologic assessment of pre- and on-treatment specimens for predicting response to anti-PD-(L)1 therapy

**Fig. 2:**
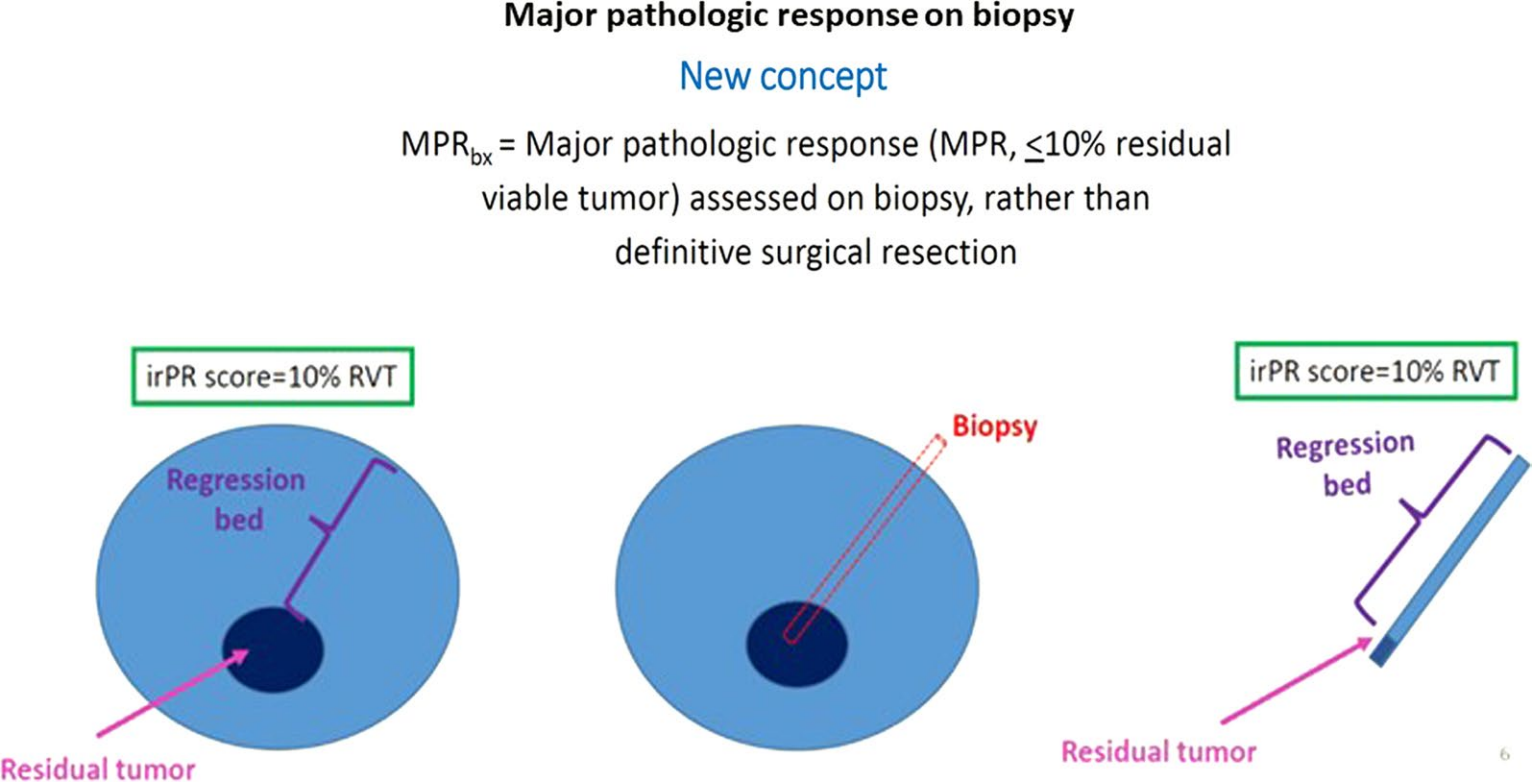
Major pathologic response assessed on biopsy, rather than definitive surgical resection

Anti-PD-(L)1-based therapies have shown remarkable effects in patients with advanced cancers, and may also improve outcomes for patients with resectable cancers. The neoadjuvant setting also provides a critical window for examining pathologic response features within the resected tumor. Pathologic response is a possible surrogate for long-term outcomes, and thus examination of the definitive resection specimen for residual viable tumor (RVT) can potentially be used to predict clinical benefit after weeks or months after therapy initiation, rather than waiting a years for overall survival data to be resulted. As such, it can be used to help guide therapeutic adjustments on both an individual patient-level and immunotherapy field-level. Numerous clinical trials for neoadjuvant immunotherapy are underway and many of these include assessments of pathologic response (e.g. pathologic complete response [pCR], i.e. 0% RVT, or major pathologic response [MPR] i.e. ≤ 10% RVT) as proposed endpoints. Scoring of pathologic response was first developed for neoadjuvant chemotherapy. Chemotherapy and immunotherapy have distinct mechanisms of action and thus, they have distinct histologic features of response, necessitating the development of a new scoring system. To meet that need, Cottrell, et al. developed a proposal for immune-related pathologic response criteria (irPRC) using hematoxylin and eosin-stained slides from the definitive resection specimens and routine light microscopy [43]. Features of immune-mediated tumor regression included dense tumor infiltrating lymphocytes, foamy macrophages, and tissue repair (neovascularization and proliferative fibrosis), amongst others. Specific attention was paid to ensure that the histologic areas studied for features of residual viable tumor or evidence of immune-mediated regression were correlated with the pre-resection radiographic ‘tumor’ measurements. The proposed irPRC was then tested for interobserver reproducibility amongst 5 pathologists and showed very high reproducibility at 10% intervals of residual viable tumor with an interclass coefficient > 0.9 [43]. Similar histologic features have since been reported in specimens from patients with melanoma and many other solid tumor types that were treated with anti-PD-1-based therapies in the neoadjuvant setting [43]. The main goal in the development of such a histology based scoring system is to predict long-term survival after only a few weeks on therapy. The data in the neoadjuvant setting is not yet mature enough to correlate the observed histologic features with 5 year patient outcomes. However, these same histologic features are observed in immune-mediated tumor regression in patients with advanced disease treated with anti-PD-1 based therapy, where data on long-term follow up is available. To test the association of these features with survival, irRPC was used to score pathologic response in early on-treatment biopsy specimens (taken 2–4 weeks after therapy initiation) from patients with melanoma. Patients with a major pathologic response on biopsy (MPRbx; < 10% residual viable tumor) showed significantly improved 5-year overall survival, compared to those with a larger proportion of RVT [43] (Fig. 2) Ongoing analyses include in-depth assessment of individual histologic features observed in these hematoxylin and eosin-stained specimens, as well as clinically meaningful thresholds of RVT beyond pCR/MPR in on-treatment specimens. Taken together, these findings lend support to the idea that irPRC will be predictive of OS in the neoadjuvant setting. They also suggest that early on-treatment H&E biopsies for patients advanced setting may have clinical utility in indicating a response to therapy, weeks before radiographic evidence is present.

## Session—emergent strategies

### Promising treatment strategies in early development

Interleukin-1 (IL-1) has been known to be a key mediator of immunity and inflammation. Its dysregulation has been implicated in tumorigenesis and tumor progression, and its upregulation is thought to be associated with many tumors. Overexpression of the IL-1 agonists IL-1a and IL-1b has been shown to promote tumor invasiveness and metastasis by inducing the expression of angiogenic genes and growth factors. Tumor IL-1 signaling is also involved in resistance to immunotherapy and immune evasion with robust evidence in NSCLC and pancreatic cancer. IL-1 receptor accessory protein (IL1RAP) is required to activate IL-1 receptor signaling. IL1RAP is expressed in several solid tumors, both on cancer cells and tumor-associated inflammatory cells. IL-1β blockade with canakinumab significantly reduced incidence of lung cancer in the CANTOS trial [44]. CAN04 (nidanilimab) is a humanized IgG1 monoclonal antibody targeting IL1RAP, blocking IL-1α and β signaling and triggering antibody-dependent cellular cytotoxicity (ADCC). Preclinical data in a NSCLC patient-derived xenograft model showed synergistic effects between CAN04 and cisplatin/gemcitabine. In a phase IIa trial, CAN04 was well tolerated with infusion-related reactions the most frequent adverse event in patients with various solid tumors [45]. A recommended phase 2 dose of 10 mg/kg was established. The dose expansion phase of the trial will evaluate CAN04 as monotherapy as well as in combination with cisplatin/gemcitabine in NSCLC and gemcitabine/nab-paclitaxel in pancreatic ductal adenocarcinoma.

The STING pathway senses intracellular DNA, triggering an immediate production of type I IFN. STING activation has wide-ranging impact on both the innate and adaptive immune response by inducing antigen-presenting cell recruitment and priming CD8+ T cells against tumor antigens. MIW815 (ADU-S100) is a synthetic cyclic dinucleotide (CDN), a first-in-class STING agonist. MIW815 (ADU-S100) has shown efficacy in combination with PD-1 checkpoint inhibitors and elicited near complete clearance of injected and non-injected tumors in mice. In a phase 1b combination trial of intratumorally administered MIW815, a novel synthetic cyclic dinucleotide that activates the STING pathway, and spartalizumab, a humanized IgG4 monoclonal antibody that blocks the binding of PD-1 to PD-L1/2 [46]. Data from 83 patients with advanced/metastatic solid tumors or lymphoma were evaluated. The most common primary diagnoses were melanoma (42.2%) and triple-negative breast cancer (13.3%). Most patients (72.3%) received prior immunotherapy. No dose-limiting toxicities were reported in any of the dosing cohorts and adverse events with the combination were no more frequent or severe than those reported with either single-agent. Systemic IFN-β levels appeared to increase in a dose-dependent manner and a trend was observed between IFN-β levels and systemic exposure. Other cytokines detected (IP-10, MCP-1, and IL-6) did not demonstrate significant dose dependency and/or PK/PD relationships. CD8 frequency as detected by IHC increased in responding patients with high PD-L1 at baseline. The combination of MIW815 and spartalizumab demonstrated antitumor activity in PD-1-naïve patients with TNBC and patients with anti PD-1-relapsed/refractory melanoma. Five patients in the MIW815 weekly-dose cohort achieved confirmed responses, including one complete response. Three of these responses, including the complete response, occurred among patients with immunotherapy-naive triple-negative breast cancer, two of whom had PD-L1 expression greater than 1% at baseline. The other two responders had previously received immunotherapy. These findings are promising as the combination was well-tolerated, with no dose-limiting toxicities reported to date and demonstrated anti-tumor activity.

### Adjuvant therapy for high-risk melanoma—current status

Most patients with stage III (lymph node positive) melanoma are offered adjuvant therapy, although if only one node is involved with very small amounts of melanoma (< 1 mm) some centers elect observation. Ipilimumab was the first successful checkpoint inhibitor in metastatic melanoma and has also been shown to be effective in the adjuvant setting. However, adjuvant treatment with ipilimumab is associated with significant toxicity, especially since the adjuvant dose is three-fold higher than the metastatic dose. The toxicity of ipilimumab indicates that nivolumab and pembrolizumab may be preferred as adjuvant treatment. In the CheckMate-238 trial, the 1-year rate of RFS was 70.5% with nivolumab versus 60.8% with ipilimumab (HR for disease recurrence or death, 0.65; 97.56% CI 0.51–0.83; P < 0.001) [7]. Treatment-related grade 3–4 adverse events were reported in 14% of patients treated with nivolumab versus 46% of patients treated with ipilimumab. Similarly, pembrolizumab significantly increased 1-year rate of RFS versus placebo (HR for recurrence or death, 0.57; 98.4% CI 0.43–0.74; P < 0.001) with 15% of pembrolizumab patients experiencing treatment-related grade 3–5 adverse events [6]. Targeted therapy with BRAF/MEK inhibitor combinations are also effective as adjuvant therapy, with an estimated three-year RFS rate of 58% observed with dabrafenib plus trametinib versus 39% with placebo (HR for relapse or death, 0.47; 95% CI 0.39–0.58; P < 0.001), and so represent a new standard of care for BRAF-positive patients.

Immunotherapy with anti-PD-1 agents is effective in adjuvant therapy for all high-risk melanoma patients while BRAF/MEK targeted therapy is effective for high-risk patients with BRAF mutation. Present clinical trial-based evidence suggests that both targeted agents and anti-PD-1 agents derive clinical benefit and a tolerable profile in the adjuvant setting in BRAF-mutant patients. However, there is no long-term (> 4 years) head-to-head comparison. Targeted agents involve daily oral administration versus IV infusion every 2–4 weeks with anti-PD-1 therapy. Toxicity with BRAF/MEK inhibitors can be chronic but manageable and can be reversed by treatment interruption, while toxicity with anti-PD-1s is uncommon but can be severe and may not always be reversible.

Findings from ongoing adjuvant trials are eagerly anticipated. These include two US Intergroup studies, the E1609 trial of ipilimumab 3 or 10 mg/kg versus high-dose IFN and the S1404 trial of pembrolizumab versus high-dose IFN or ipilimumab 10 mg/kg, and the CheckMate 915 trial of combined nivolumab plus ipilimumab versus nivolumab monotherapy. Several clinical trials are ongoing, using nivolumab and pembrolizumab in monotherapy or in combination with chemotherapy, radiotherapy, other immunotherapies, and targeted therapies. Depending on the molecular features of the patients and tumors, as well as the responses to therapy, personalized treatment should be considered for melanoma patients, in order to achieve better clinical benefits. Further research is necessary to explore oncogenic pathways and the TME potential in the treatment of melanomas.

### Neoadjuvant immunotherapy—the pathway to therapy personalization

Locally/regionally advanced melanoma confers a major challenge in terms of surgical and medical management. Surgical treatment carries the risks of surgical morbidities and potential complications that could be lasting. In addition, these patients continue to have a high risk of relapse and death despite the use of standard adjuvant therapy. Neoadjuvant therapy has the potential to significantly improve the clinical outcome of these patients, particularly in this era of newer and effective targeted and immunotherapeutic agents. Several neoadjuvant targeted and immunotherapy studies have been completed in melanoma to date and have yielded promising clinical activity.

Potential benefits of neoadjuvant immunotherapy include reduction in tumor burden before surgery with improvements in surgical resectability, organ preservation, and improvement in overall survival (OS). In addition, pathological response evaluation can serve as surrogate outcome markers for RFS and OS in addition to clinical and radiologic responses, and stronger and broader tumor-specific T cell responses may be induced. Nonresponding patients however may deteriorate before potential curative surgery, which might also be impaired by immune-related adverse events.

Neoadjuvant studies provide access to blood and tumor biospecimens before and during systemic therapy, supporting studies of immunologic and histologic correlates of tumor response. Such studies can allow for better understanding of the antitumor mechanisms of action and ultimately would enable more selective application of therapeutic agents to patients who are more likely to benefit.

In the OpACIN study, neoadjuvant ipilimumab plus nivolumab did not delay surgery and the pathologic response rate was high (78%) but treatment was highly toxic with 90% grade 3–4 adverse events. Also, higher frequency of tumor resident T cell receptor (TCR) clones was observed in neoadjuvant treated tumors [47]. In the phase II OpACIN-neo trial to identify the optimal neoadjuvant combination scheme of ipilimumab and nivolumab, two cycles of ipilimumab 1 mg/kg plus nivolumab 3 mg/kg was tolerated and induced a pathological response in a high proportion of patients [48].

In a pooled analysis from the International Neoadjuvant Melanoma Consortium (INMC), neoadjuvant immunotherapy and targeted therapy showed efficacy in resectable clinical stage III melanoma patients and were associated with high pCR rate [19].

In both the OpACIN and OpACIN-neo trials, no patients with a pathologic response had relapsed after a median follow-up of 30 and 8.3 months, respectively. In stage IV melanoma, long-term benefit is observed in patients achieving CRs with immune checkpoint inhibition, even after cessation of therapy. This raises the question of whether complete lymph node dissection (CLND) can be omitted when a complete pathologic response with neoadjuvant immunotherapy is achieved. The aim of the phase II PRADO study is to confirm the pathological response rate and toxicity of neoadjuvant ipilimumab plus nivolumab. Additional aim is to determine subsequent therapy i.e. omitting surgery and adjuvant nivolumab based on the pathological response. The pathologic response in the largest lymph node can be considered a reliable indicator of therapeutic response to neoadjuvant immunotherapy in patients with stage III melanoma [49]. To date, the PRADO trial demonstrated that pre-treatment biomarker analysis in index lymph node as well as pathologic evaluation of the marked lymph node is feasible.

These results describe the feasibility of neoadjuvant immune checkpoint blockade in melanoma demonstrating that neoadjuvant immunotherapy may be superior to adjuvant immunotherapy. However, neoadjuvant may not offer benefit over adjuvant therapies to all patients because it induced high toxicity rates; therefore, it needs to be further investigated to balance efficacy and toxicity. Immune correlates of response were identified, demonstrating higher lymphoid infiltrates in responders. Personalization of neoadjuvant immunotherapy based on DNA/RNA signatures and personalization of surgical extent may become a standard.

### What combinations are really worth it?

Nivolumab plus ipilimumab show a sustained long-term benefit with 5-year OS of 52% in the CheckMate 067 trial, compared to 44% with nivolumab and 26% with ipilimumab [50]. Even after discontinuation, many patients may continue to derive benefit from combination nivolumab plus ipilimumab, with similar outcomes for patients who discontinued treatment because of toxicity during the induction phase and those who did not [50, 51]. The effect of the combination has also been explored using a novel endpoint of treatment-free survival (TFS), defined as the area between Kaplan–Meier curves for time to immune checkpoint inhibitor protocol therapy cessation and time to subsequent systemic therapy initiation or death, partitioned as time with and without toxicity [52]. This revealed a long PFS without toxicity after treatment cessation for patients who received nivolumab plus ipilimumab. Given the uncertainty of the benefit of repeated dosing of nivolumab plus ipilimumab, a de-escalation study of dropping ipilimumab after two doses of nivolumab plus ipilimumab for selected patients is being investigated (NCT03122522).

In another trial, two different combination dose regimens were assessed and showed a significantly lower incidence of treatment-related grade 3–5 adverse events with nivolumab 3 mg/kg plus ipilimumab 1 mg/kg compared with the approved doses of nivolumab 1 mg/kg plus ipilimumab 3 mg/kg [53]. No difference in PFS or OS was observed between groups, although the study was not powered to determine whether there was an efficacy difference between these two arms.

Nivolumab plus ipilimumab is also very effective in melanoma patients with brain metastases, with durable intracranial responses achieved by 51% of patients with asymptomatic brain metastases with no prior local brain therapy [54]. Two-year intracranial PFS was 49% and 2-year OS was 63%. The combination may also have a role as adjuvant therapy in patients with surgically resectable or completely irradiated stage IV disease. In the phase II IMMUNED trial in stage IV melanoma patients with resected or completely irradiated melanoma, adjuvant therapy with nivolumab alone or in combination with ipilimumab resulted in improved RFS compared placebo [55]. The rate of grade 3–4 treatment-related adverse event was higher than reported in the CheckMate 067 trial in patients with metastatic disease, but no treatment-related deaths had occurred. However, the combination of nivolumab plus ipilimumab may not be better than nivolumab monotherapy as adjuvant therapy for stage III resectable disease (https://news.bms.com/press-release/corporatefinancial-news/bristol-myers-squibb-announces-update-checkmate-915-opdivo-niv).

### Therapeutic targets in non-T cell-inflamed tumors

Responses to immunotherapy preferentially occur in tumors with a pre-existing antitumor T-cell response that can be measured by expression of DC and CD8+ T cell-associated genes. The tumor subset with this signature has been described as the T cell-inflamed phenotype. Understanding mechanisms of resistance in non-inflamed tumors will help address treatment failure and increase the proportion of patients responding to immunotherapy.

Intrinsic β-catenin signaling contributes to a lack of T-cell infiltration in melanoma. β-catenin represses CCL4, leading to lack of Batf3+ DC recruitment, failed T cell priming, and lack of response to checkpoint blockade. There is an inverse correlation between WNT/β-catenin and T cell-inflamed signature using transcriptional analysis across tumor types [56]. Gene expression signatures associate with resistance to immune-checkpoint inhibition and mutations associated with T cell-inflamed or non-T cell-inflamed phenotypes have been clinically validated. For example, one gene signature, the Tumor Inflammation Signature (TIS), has been developed as a clinical-grade assay that provides both quantitative and qualitative information about the immune environment within a tumor, reporting on the presence of an immune infiltrate as well as the functional status of T cells. The TIS, developed on the NanoString nCounter^®^ gene expression system (NanoString Technologies, Inc., Seattle, WA, USA), is an 18-gene signature that measures the status of adaptive immune response within the tumor. The TIS contains IFN-γ-responsive genes related to antigen presentation, chemokine expression, cytotoxic activity, and adaptive immune resistance.

Tumor mutation load or tumor mutation burden (TMB), measured by comprehensive genomic profiling, is an important emerging biomarker that shows promise in its ability to predict the response to immune checkpoint inhibitors. TMB is a measure of the number of mutations within a tumor genome, defined as the total number of mutations per coding regions. There is large variability in TMB within tumor types, ranging from just a few to thousands of mutations. A high TMB load has been shown to be associated with better response rates to checkpoint inhibitors in melanoma, NSCLC, and urothelial carcinoma. Limited responses to anti-PD-1 and anti-PD-L1 agents are associated with tumor types exhibiting low TMB such as colorectal, ovarian, and prostate tumors.

Targeting of these mutations or pathways may present rational immunotherapy combination approaches to overcome non-T cell-inflamed tumors or augment immunotherapy in T cell-inflamed tumors and multiple clinical trials are being planned and initiated. For example, mutations in the isocitrate dehydrogenase (IDH)-1 and IDH2 genes may facilitate escape from immune surveillance in a subset of malignant gliomas. Reduced expression of CTLA genes and IFN-γ-inducible chemokines has been shown in IDH-mutated tumors. Treatment with IDH-C35, a selective mutant IDH-1 inhibitor, restored chemokine expression, promoted T-cell infiltration, and increased the efficacy of therapeutic peptide vaccination against IDH-mutated gliomas in mice [57]. The combination of an IDH1 inhibitor (ivosidenib) plus nivolumab is now being investigated in a phase II study in patients with IDH1-mutant advanced solid tumors. Novel clinical-grade biomarkers are needed to guide the choice of the immunotherapy agents and combinations to obtain the maximal likelihood of patient benefit. Translational endpoints include changes in IFN-associated gene expression, epigenetic and metabolomics changes in tumor and tumor infiltrating and peripheral blood immune cells and ctDNA.

### Novel circulating biomarkers in melanoma

**Fig. 3:**
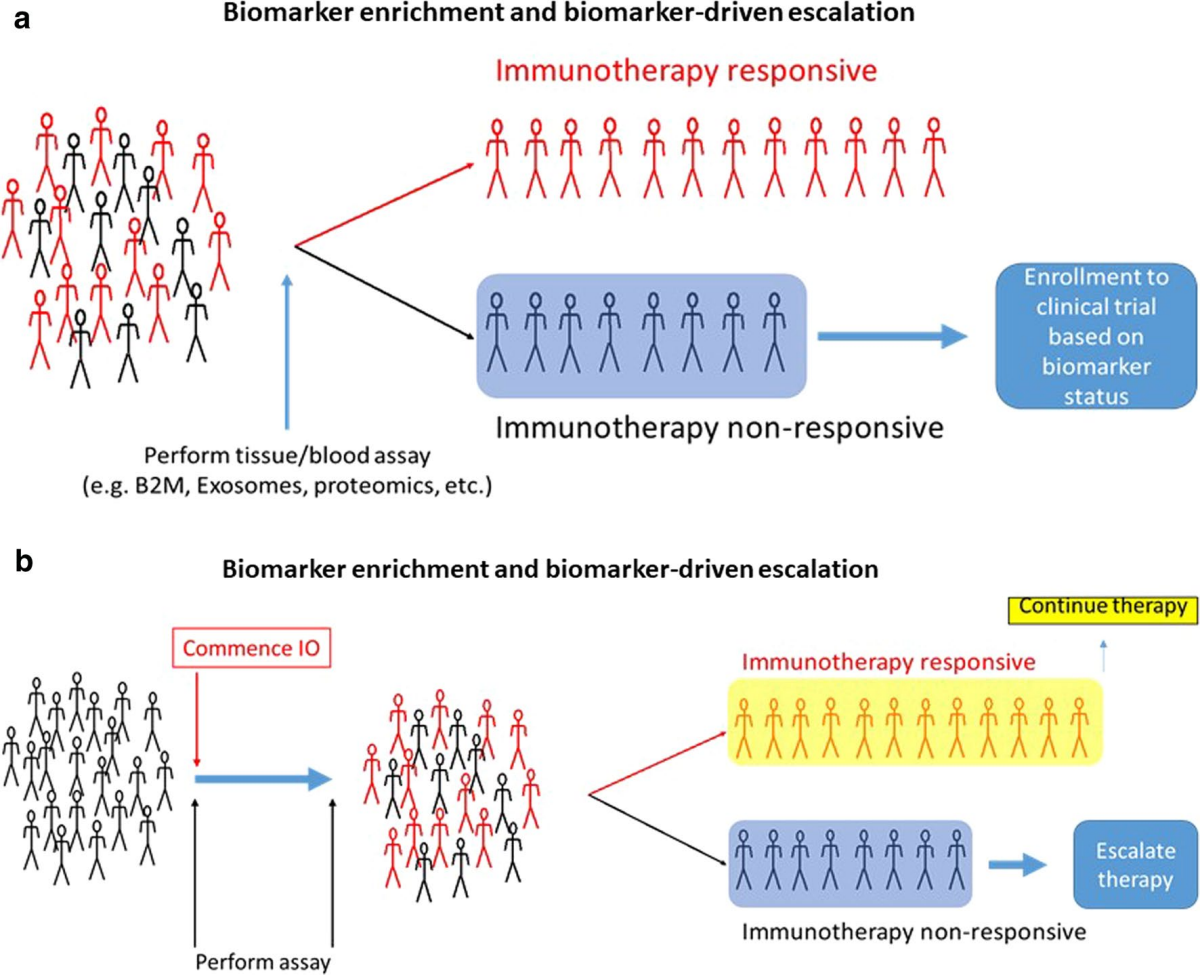
Biomarker enrichment and biomarker-driven escalation. **a** Biomarker enrichment (current strategy—PD-L1 in NSCLC). **b** Biomarker-directed escalation

Predictive biomarkers are needed to help optimally select patients for adjuvant therapy or treatment of metastatic disease, to monitor response and recurrence, to better understand mechanisms of resistance, and to identify patients at risk of severe toxicity. Biomarkers study can be used to help develop next generation immunotherapy agents and combinations treatments for melanoma. In biomarker enrichment (Fig. 3a), tissue or blood assays can be used to identify immuno-responsive patients (e.g. based on PD-L1 status) and non-responsive patients, who may be enrolled into trials based on biomarker status. Biomarker-directed escalation (Fig. 3b) involves performing assays after initiation of immunotherapy in order to identify which patients are responsive and should continue therapy and which do not respond and may need therapy escalated.

Whole plasma and exosome proteomic profiling may be used to develop a predictive model of immunotherapy response and toxicity, and to obtain insight into the mechanisms underlying immunotherapy resistance. In a cohort of 150 melanoma patients receiving anti-PD-1 antibodies whole plasma was analyzed at baseline, and on-treatment at 6-week and 6-month timepoints [58]. Proteomic analysis in plasma was performed using a multiplex proximity extension-based assay that enabled simultaneous detection of more than 1000 proteins including cytokines, chemokines and growth factors. Patients who responded to immunotherapy vs those who did not respond had differentially expressed proteins at baseline, including interleukin (IL) 33 receptor (ST2), IL-6, CCL13 and stem cell factor (SCF) that were predictive of OS and progression-free survival (PFS). Whereas high baseline and on-treatment levels of IL-6 have been associated with worse survival in clinical trials of nivolumab alone or in combination with ipilimumab [31, 59]. Considering the biomarker-directed escalation, 70 differentially expressed cytokines and chemokines were identified between baseline and on-treatment time-points, the majority of which were reflective of immune activation. In addition, many more differentially expressed proteins were identified between responders and non-responders at 6 weeks than at baseline. Several 6-week differentially expressed proteins were predictive of survival (e.g. Inducible T Cell Costimulatory Ligand (ICOSLG), IL-8 and MIA). However, it is too early to consider using these biomarkers in escalation trials and further validation is ongoing.

## Conclusions

Recent insights into genetic and phenotypic characterization of specimens from patients with melanoma have initiated a new era of rapidly evolving treatments. The use of novel immunotherapies, especially immune checkpoint inhibitors including CTLA-4, PD-1 and others, as well as targeted BRAF and MEK inhibitors have significantly improved outcomes for many patients with metastatic melanoma. However, despite these improvements, most patients still either fail to respond or will relapse over time. There is a need to increase the ratio of patients who benefit from the major advances in therapy vs those who did not benefit. Better understanding of the TME and host immune response may lead to development of biomarkers that help identify patients for the best treatment option, as well as new treatments and new combination strategies. Increased use of these new systemic treatments in the neoadjuvant and adjuvant settings may also help improve long-term outcomes for patients.

## Data Availability

Not applicable.

## Abbreviations

ACT: Adaptive cell transfer
ADP: Adenosine diphosphate
ATP: Adenosine triphosphate
AJCC: American Joint Committee on Cancer
AMP: Adenosine monophosphate
CAR-T: Chimeric antigen receptor T
CCL4: C–C motif chemokine ligand 4
CDK: Cyclin-dependant kinase
CIDC: Cancer Immunologic Data Commons
CIMAC: Cancer Immune Monitoring and Analysis Centers
CLND: Completion lymph node dissection
CR: Complete response
CSPG4: Chondroitin sulfate proteoglycan 4
ctDNA: Cell free tumor DNA
CTLA-4: Cytotoxic T-lymphocyte-associated antigen
CyTOF: Mass cytometry by time of flight
DC: Dendritic cell
DFS: Disease free survival
DNA: Deoxyribonucleic acid
DNR: Do-not-resuscitate
ECT: Electrochemoterapy
EP: Electroporation
FDA: Food and Drug Administration
FMT: Fecal microbial transplant
FNIH: Foundation of National Institutes of Health
GBM: Glioblastoma multiforme
GEP: Gene expression profile
HAVCR2: Hepatitis A virus cellular receptor 2
ICI: Immune checkpoint inhibitor
ICOSLG: Inducible T Cell Costimulatory Ligand
IDO1: Indoleamine
IFN: Interferon
IgG: Immunoglobulin G
IL: Interleukin
INMC: International Neoadjuvant Melanoma Consortium
IO: Immune oncology
IR: Inhibitor receptor
LAG3: Lymphocyte activation gene 3
LDH: Lactate dehydrogenase
MDSCs: Myeloid derived suppressor cells
MHC: Major histocompatibility complex
mIF: Immunofluorescence
mIHC: Multiplex immunohistochemistry
MPR: Major pathologic response
MSS: Melanoma-specific survival
NAD: Adenine dinucleotide
NCI: National Cancer Institute
NGFR: Nerve growth factor receptor
NIH: National Institutes of Health
NSCLC: Non small cell lung cancer
NYU: New York University
OR: Odds ratio
ORR: Overall response rate
OS: Overall survival
PACT: Accelerating cancer therapies
pCR: Pathologic complete response
PD-1: Programmed death-1
PD-L1: Programmed death ligand-1
PET: Positron emission tomography
PFS: Progression-free survival
PI3K: Phosphotidylinosital 3-kinase
PK: Pharmacokinetic
PR: Partial response
PTEN: Tumor suppressor phosphatase and tensin homolog
PTPN2: Protein tyrosine phosphatase non-receptor type 2
RCC: Renal cell carcinoma
RFS: Recurrence free survival
RNA: Ribonucleic acid
RVT: Residual viable tumor
scFv: Single chain variable fragment
SD: Stable disease
SNLB: Sentinel lymph node biopsy
SOP: Standard operating procedure
STING: Stimulator of interferon genes
TAA: Tumor-associated antigen
TCGA: The Cancer Genome Atlas
TCR: T cell receptor
TEX: Tumor derived exosome
TGFβ: Transforming growth factor β
TIGIT: T cell tyrosine-based inhibitor
TILs: Tumor-infiltrating lymphocytes
TMaE: Tumor macroenvironment
TMB: Tumor mutation burden
TME: Tumor microenvironment
TPS: Tumor proportion score
trAE: Treatment-related adverse event
T-Vec: Talimogene laherperepvec
UM: Uveal melanoma
UV: Ultra-violet
WNTβ: Wingless integrated
